# UNS S31603 Stainless Steel Tungsten Inert Gas Welds Made with Microparticle and Nanoparticle Oxides

**DOI:** 10.3390/ma7064755

**Published:** 2014-06-20

**Authors:** Kuang-Hung Tseng, Po-Yu Lin

**Affiliations:** Institute of Materials Engineering, National Pingtung University of Science and Technology, No. 1, Hseuhfu Rd., Neipu, Pingtung 91201, Taiwan; E-Mail: m10145003@mail.npust.edu.tw

**Keywords:** microparticle, nanoparticle, activated oxide, thermal stability, particle size

## Abstract

The purpose of this study was to investigate the difference between tungsten inert gas (TIG) welding of austenitic stainless steel assisted by microparticle oxides and that assisted by nanoparticle oxides. SiO_2_ and Al_2_O_3_ were used to investigate the effects of the thermal stability and the particle size of the activated compounds on the surface appearance, geometric shape, angular distortion, delta ferrite content and Vickers hardness of the UNS S31603 stainless steel TIG weld. The results show that the use of SiO_2_ leads to a satisfactory surface appearance compared to that of the TIG weld made with Al_2_O_3_. The surface appearance of the TIG weld made with nanoparticle oxide has less flux slag compared with the one made with microparticle oxide of the same type. Compared with microparticle SiO_2_, the TIG welding with nanoparticle SiO_2_ has the potential benefits of high joint penetration and less angular distortion in the resulting weldment. The TIG welding with nanoparticle Al_2_O_3_ does not result in a significant increase in the penetration or reduction of distortion. The TIG welding with microparticle or nanoparticle SiO_2_ uses a heat source with higher power density, resulting in a higher ferrite content and hardness of the stainless steel weld metal. In contrast, microparticle or nanoparticle Al_2_O_3_ results in no significant difference in metallurgical properties compared to that of the C-TIG weld metal. Compared with oxide particle size, the thermal stability of the oxide plays a significant role in enhancing the joint penetration capability of the weld, for the UNS S31603 stainless steel TIG welds made with activated oxides.

## 1. Introduction

Although tungsten inert gas (TIG) welding can produce a high-quality weld, its application is usually limited to thin plates welded by a single-pass procedure, and the process also suffers from relatively low productivity. The low productivity of TIG welding results from a combination of a low deposition rate and shallow joint penetration. TIG welding can increase in potential if the productivity can be improved significantly. Increasing the productivity provides a significant economic benefit to the process of fabrication by welding, in terms of time and cost savings. One approach to increase the productivity of TIG welding is to add quantities of minor surface-active elements to the molten metal. This can be accomplished by a number of technologies, including coated activated fluxes (such as oxides or sulfides) on the steel plate surface [1,2,3,4,5,6,7,8,9,10] or added active gases (such as oxygen, carbon dioxide or sulfur dioxide) to an inert gas [11,12,13]. The TIG welding with activated flux is a notable arc welding that can dramatically increase the joint penetration. This variant of TIG welding, called activated TIG (A-TIG) welding, was proposed by the E.O. Paton Electric Welding Institute of the NAS of Ukraine [14]. A-TIG welding intensifies the conventional TIG (C-TIG) welding process, allowing for the joining of thick plates by a single-pass operation without edge preparation and filler metal, instead of using multi-pass welding procedures. Moreover, the heat-to-heat variations in weld depth for some stainless steels or nickel-based alloys can be eliminated by using an activated flux. Thus, this method of improving both the amount and consistency of the joint penetration in the TIG weld may provide a significant economic benefit to the manufacturing industry.

A key factor in A-TIG welding is the composition of an activated flux. The flux is a mixture of activated compounds suspended in a carrier solvent. The most common activated compounds include oxides, fluorides and chlorides, among many others, and are generally varied depending on the type of base metal to be welded and the particular welding process to be used. The constant characteristic of an activated flux is that for the flux to perform its function, it must be evenly spread over the entire steel plate surface to be welded. Tseng *et al.* [4,5,6] investigated the effect of different activated fluxes on the weld morphology of stainless steel and concluded that the microparticle SiO_2_, TiO_2_, MoO_3_ and Cr_2_O_3_ produced a deep, narrow weld, whereas the microparticle Al_2_O_3_ caused the deterioration in weld depth and bead width compared with the C-TIG weld. Tseng and Chuang also reported that the microparticle FeO and FeS provided a high depth-to-width (D/W) ratio of the stainless steel TIG weld, whereas the microparticle FeF_2_ did not significantly increase the weld D/W ratio [7]. Furthermore, Tseng and Wang reported that the TIG welding with an activated flux composed of the microparticle FeS/FeF_2_ has the potential advantages of a satisfactory surface appearance, full joint penetration and less angular distortion for stainless steel TIG weldment [10]. The quantity of the flux-coated layer is also a key factor in A-TIG welding. The weld depth initially increases sharply with coated quantity, before becoming approximately constant and subsequently decreasing [8]. Rückert *et al.* [15] reported that the optimal coated thickness of the microparticle SiO_2_ depends on the welding current. They observed that the maximum depth of the TIG weld was obtained with a coated thickness of 40 μm at a welding current of 100 A and at a thickness of 70 μm at a current of 150 A.

In studies of the TIG welding with activated compound, microparticle oxides were commonly used as the activated compounds. To improve the performance of A-TIG welding, the effects of the thermal stability and particle size of activated oxides on the A-TIG weld characteristics must be ascertained. Such data are also important in enhancing the joint penetration capability of the A-TIG weld. In addition, it is necessary to choose a suitable solvent, resulting in a homogeneous distribution of the flux-coated layer. This study also investigated the relationship between the activated compounds and polar solvents in A-TIG welding. Two kinds of powdered compound were used to investigate the effects of the thermal stability and particle size of the activated oxides on the surface appearance, geometric shape, angular distortion, delta ferrite content and Vickers hardness of the stainless steel TIG weld. Three kinds of polar solvent were also used to investigate the coverability, spreadability and volatility of the flux-coated layer.

## 2. Experimental Details

UNS S31603 stainless steel with the chemical composition listed in Table 1 was used in this study. Its chemical composition was determined by a glow discharge spectrometer (GDS-750 QDP, LECO, St. Joseph, MI, USA). Experimental analysis was performed by the Instrumentation Center at National Cheng Kung University. The steels were cut into strips with dimensions of 100 mm in length and 100 mm in width for 6 mm thick plates. Before welding, all steel plates were roughly ground with 240 grit SiC sandpaper to remove surface impurities and then cleaned with acetone.

**Table 1:** Chemical composition of UNS S31603 stainless steel (wt%, balance Fe).

Specification	Chemical composition (wt%)							
	C	Si	Mn	P	S	Cr	Ni	Mo
ASTM A240	0.03 max	1.00 max	2.00 max	0.045 max	0.030 max	16.0–18.0	10.0–14.0	2.00–3.00
GDS	0.02	0.45	1.69	0.028	0.016	16.6	10.8	2.11

**Figure 1:**
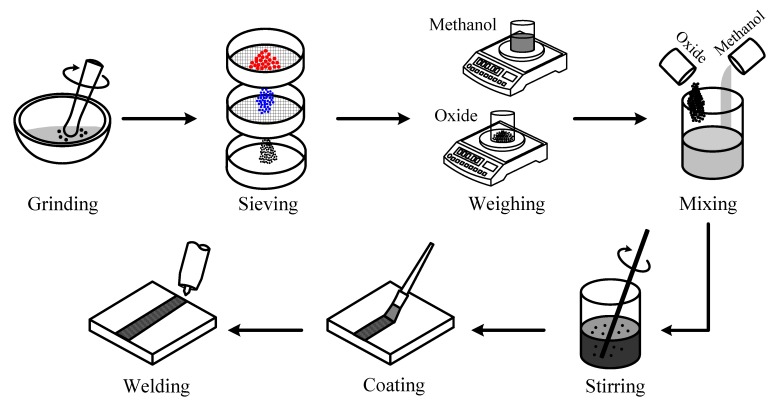
Preparation procedures of activated tungsten inert gas (A-TIG) welding.

SiO_2_ and Al_2_O_3_ were used as the activated compounds in this study. Table 2 shows the particle size and mass density of SiO_2_ and Al_2_O_3_. Figure 1 shows the preparation procedures of A-TIG welding, which include the oxide grinding, oxide sieving, oxide/solvent weighing, oxide/solvent mixing, oxide/solvent stirring, flux coating and TIG welding. In A-TIG welding, the oxide was mixed with a methanol and stirred with a glass rod until the mixture attained a paint-like consistency. The flux paste was then manually applied onto the steel plate surface with a 12 mm-wide paintbrush. The methanol was allowed to evaporate, leaving a thin flux-coated layer attached to the surface of the steel plate. The coated layer had a width and length of 12 mm and 100 mm, respectively. The weight per unit area of the coated layer was approximately 2.26 ± 0.21 mg/cm^2^. The exposure time of the coated layer before welding was 3.5 min. The basis for the selection of the flux coating parameters formulated by the authors on the basis of A-TIG welding trials was confirmed by past results.

Welding was carried out by using a mechanized system with constant current power supply in which a water-cooled torch was moved at a constant speed. The welding machine was conducted using direct current straight polarity (electrode negative) with a 3.2 mm diameter, 2% thoriated tungsten rod (AWS classification EWTh-2). All autogenous, single-pass, bead-on-plate TIG welds were performed under identical conditions (welding current of 180A, travel speed of 140 mm/min and electrode gap of 2 mm). The electrode tip had an included angle of 60°. A high-purity (99.99%) argon gas was used as the shielding gas with a flow rate of 12 L/min. The gas cup had a diameter of 12.8 mm. The included angle of the electrode tip was ground, and the electrode gap was measured for each new weld prior to welding to ensure that all experiments were performed under equally controlled conditions. During welding, a charge-coupled device (CCD) video camera having a 480 frames/s sampling rate was used to continuously collect images of the arc profile.

**Table 2:** Particle size and mass density of activated oxides used in this study.

Properties	SiO_2_		Al_2_O_3_	
	Microparticle	Nanoparticle	Microparticle	Microparticle
Particle size	75 μm	40 nm	95 μm	50 nm
Mass density	2.32 g/cm^3^	2.15 g/cm^3^	4.00 g/cm^3^	3.84 g/cm^3^

**Figure 2:**
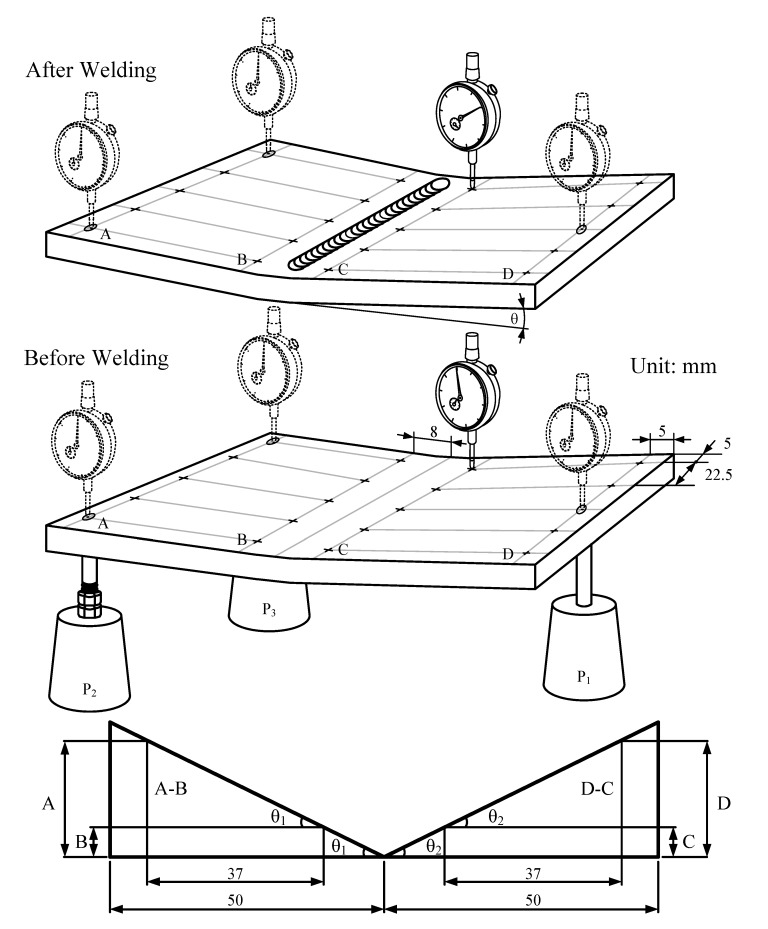
Measurement of angular distortion in bead-on-plate weldment.

During welding, the steel plates were constraint-free to avoid the influence of constrained force. Following welding, experiments were conducted to measure the angular distortion in a bead-on-plate weldment. Measurements were taken using a dial indicator having a minimum reading of 0.001 mm. The weld-induced deformations were measured using the vertical displacement method, as shown in Figure 2. A hole was drilled from the back at positions P_1_, P_2_ and P_3_, and a pillar was inserted into each hole. These three pillars (one fixed, two adjustable) were used to adjust the horizontal position of the steel plate, and the displacement from each measurement point to the horizontal surface was then recorded. This set of measurements was then normalized with reference to a zero datum. The vertical displacement measurements were taken prior to welding and again after welding at the same points. The differences in measurements prior to welding and after welding revealed the vertical displacement and indicated the degree of the resulting deformations of the steel plate due to the thermal load caused by welding. The total angular distortion (θ) was derived using the following equation:

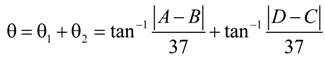
(1)


A feritscope (FMP30, Fischer, Germany) was used to measure the ferrite number (FN) of the UNS S31603 stainless steel weld metal. Samples for ferrite content measurement were prepared as follows: first, the surface was ground with 400 grit SiC sandpaper to remove slag. A probe was then placed in direct contact with the weld metal. To minimize measurement errors resulting from inhomogeneity in the weld metal, the average value of seven measurements taken from various locations along the weld metal was calculated.

An oxygen/nitrogen/hydrogen analyzer (ONH836, LECO, St. Joseph, MI, USA) was used to measure the oxygen content in the stainless steel weld metal. Samples for oxygen content measurement were prepared as follows: first, the surface was ground with 400 grit SiC sandpaper to remove slag. The weld metal was then cut out directly for use as an analytical specimen.

The surface appearance and geometric shape of the weld were photographed with a metallographic microscope. All joints were cross-sectioned perpendicular to the welding direction for metallographic analyses. Samples were then prepared using standard procedures, including sectioning, mounting and grinding, as well as polishing to a 0.05 μm finish, followed by etching. The etching was carried out in an electrolyte solution consisting of 10 g of oxalic acid and 100 mL of water. The metallographic samples were examined with a Toolmaker’s microscope (TM-510, Mitutoyo, Japan) to measure the depth and width of the weld. The heat-affected zone (HAZ) was measured at different positions in a weld cross- sectional profile. Each datum represents the average of three samples or positions for a given weld. Moreover, the microhardness test was used to evaluate the microstructural features of the weldment. The hardness profile across the weld metal, HAZ and unaffected base metal was measured under a load of 2.94 N for 15 s.

## 3. Results and Discussion

The purpose of this study was to demonstrate that the surface appearance, geometric shape, angular distortion and metallurgical properties of the UNS S31603 stainless steel A-TIG welds greatly depend on the thermal stability and particle size of the activated oxides. In addition, the influence of polar solvents on the coverability, spreadability and volatility of the flux-coated layer were investigated.

### 3.1. Evaluation of Activated Oxides Mixed with Polar Solvents

**Figure 3:**
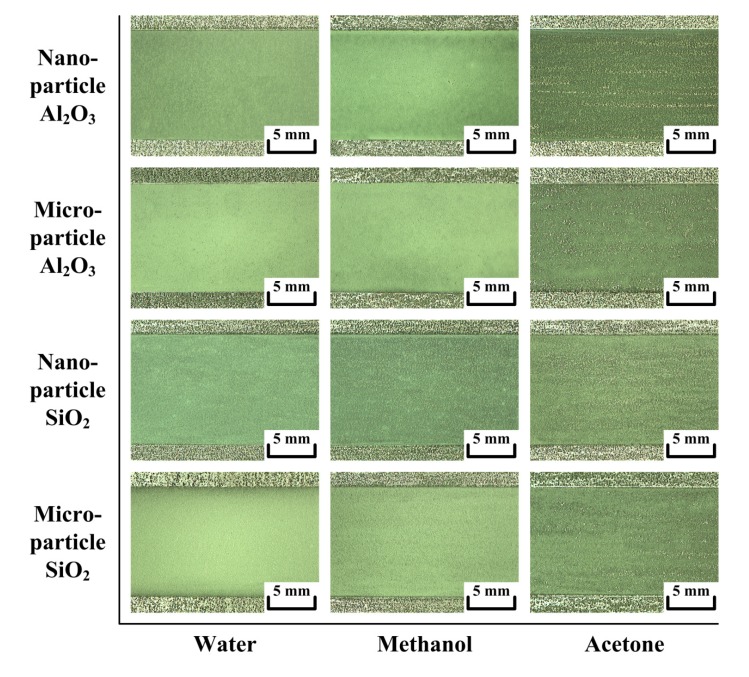
Evaluation of activated oxides mixed with polar solvents.

It is necessary to choose a suitable solvent resulting in a homogeneous distribution of the flux-coated layer for A-TIG welding. This is because the A-TIG weld appearance becomes irregular when there is an inconsistent quantity of the flux-coated layer. In this study, water, methanol and acetone were used as carrier solvents. The differences in weight concentration for microparticle and nanoparticle fluxes were used in the trial after specific surface areas were considered. Although the weight concentrations of the fluxes are different, the quantity of the flux-coated layer is near constant. In this study, 500 mg of microparticle oxide was mixed with 0.75 mL of solvent, while 250 mg of nanoparticle oxide was mixed with 4.5 mL of solvent to form a paint-like consistency. The results in Figure 3 clearly show that SiO_2_ and Al_2_O_3_ mixed with water or methanol provides a homogeneous distribution of the coated layer, while acetone is the worst solvent in terms of coated layer consistency. Selecting a carrier solvent involves the evaluation of many factors. The two most important factors are the coatability and the volatility of the flux-coated layer. The coatability can be further characterized by the spreadability and coverability. Table 3 lists the physical properties of water, methanol and acetone [16]. The dielectric constant and dynamic viscosity of water and methanol are substantially higher than those of acetone. SiO_2_ and Al_2_O_3_ mixed with water or methanol and then applied to a steel plate surface provide good spreadability and coverability of the coated layer. In A-TIG welding, the evaporation rate of the carrier solvent also plays an important role, because the solvent needs to leave the flux at an ideal rate to obtain a dry coated layer. Table 4 shows the volatile time of the activated fluxes. The results show that water takes the longest time to evaporate from the flux-coated layer, whereas acetone has an evaporation time shorter than that of methanol. Compared with methanol and acetone, water has the highest boiling point and the lowest vapor pressure, leading to poor volatility after flux coating. For powdered SiO_2_ and Al_2_O_3_, methanol is the preferred solvent in an activated flux, resulting in a good coatability, volatility of the coated layer. Compared with microparticle SiO_2_ and Al_2_O_3_, nanoparticle SiO_2_ and Al_2_O_3_ mixed with water or methanol provides good flux coverability of the coated layer. However, this is accompanied by poor flux spreadability of the layer, because the nanoparticles have a strong tendency to cluster, forming an agglomerate state [17].

**Table 3:** Physical properties of polar solvents used in this study.

Properties	Polar Solvent		
	Water	Methanol	Acetone
Dielectric constant	80.1	33.0	21.0
Dynamic viscosity (mPa·s)	0.89	0.54	0.31
Boiling point (°C)	100.0	64.6	56.1
Vapor pressure (kPa)	3.2	16.9	30.8

**Table 4:** Evaporation time of activated fluxes (at an operating temperature of 25 °C).

Activated Oxide	Polar Solvent		
	Water	Methanol	Acetone
Microparticle SiO_2_	31.78 min	1.72 min	0.45 min
Nanoparticle SiO_2_	61.13 min	3.13 min	1.28 min
Microparticle Al_2_O_3_	24.22 min	1.30 min	0.33 min
Nanoparticle Al_2_O_3_	41.35 min	2.12 min	0.87 min

### 3.2. Effect of Microparticle or Nanoparticle Oxide on Surface Appearance of A-TIG Weld

**Figure 4:**
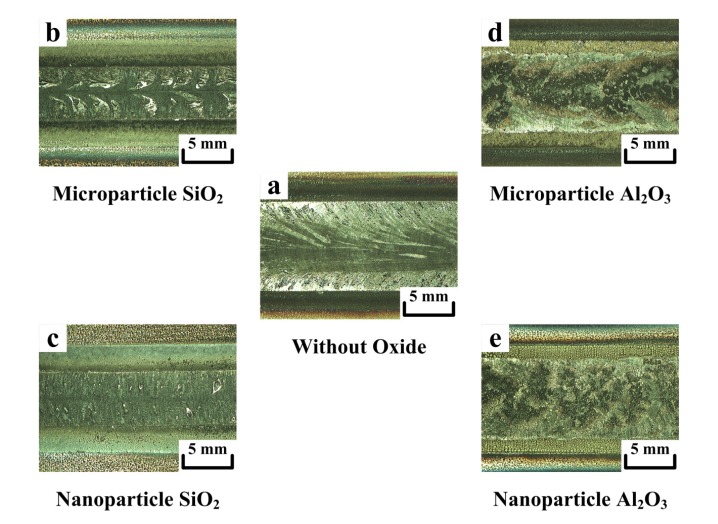
Surface appearance of TIG welds made with and without oxides.

A-TIG welding has the potential to substantially increase productivity [18]. However, industry has been slow to exploit the benefits from the increased joint penetration, partially because A-TIG welding tends to produce excessive flux slag and a rough surface compared with C-TIG welding. The flux forms slag that is less dense than the molten metal, causing the slag to float up to the weld surface. The surface appearance of the weld will be a special consideration for some manufactured products. Producing a clean, smooth weld surface is thus a key competitive challenge for the adoption of A-TIG welding in industrial applications. In this study, microparticle oxide was mixed with methanol at a weight ratio of 1:1.2; nanoparticle oxide was mixed with methanol at a weight ratio of 1:14.3. Figure 4 shows the surface appearance of the TIG welds made with and without oxides. Figure 4a shows the results of C-TIG welding, which produced the clean, smooth surface of the weld. Figure 4b shows that the microparticle SiO_2_ produced little slag (20 ± 2% surface coverage of a weld). Similarly, Figure 4c shows that the nanoparticle SiO_2_ produced little slag (10 ± 1% surface coverage of a weld). As presented in Figure 4d, a large amount of slag (40 ± 3% surface coverage) was produced when using microparticle Al_2_O_3_. In contrast, a small amount of slag (25 ± 2% surface coverage) was produced when using nanoparticle Al_2_O_3_ (Figure 4e). Compared with the surface appearance of the TIG weld made with microparticle or nanoparticle SiO_2_, the surface of the TIG weld made with microparticle or nanoparticle Al_2_O_3_ showed more particles of unmelted powder remaining on both of its sides. The melting point of SiO_2_ (1723 °C) is lower than that of Al_2_O_3_ (2100 °C) [19]. The SiO_2_ could thus have been easily melted by the TIG arc heat. A bulk material has a fixed melting point regardless of its size. However, small particles exhibit a melting point dependent on the particle size [20]. The melting point of nanoparticles is always lower than that of the corresponding microparticles, because ultrafine particles have a high proportion of surface atoms [21]. Thus, the nanoparticle oxides could have been melted by the welding arc heat more easily. The surface appearance of the TIG weld made with nanoparticle oxide has less flux slag compared with the one made with microparticle oxide of the same type.

### 3.3. Effect of Microparticle or Nanoparticle Oxide on Geometric Shape of A-TIG Weld

**Figure 5:**
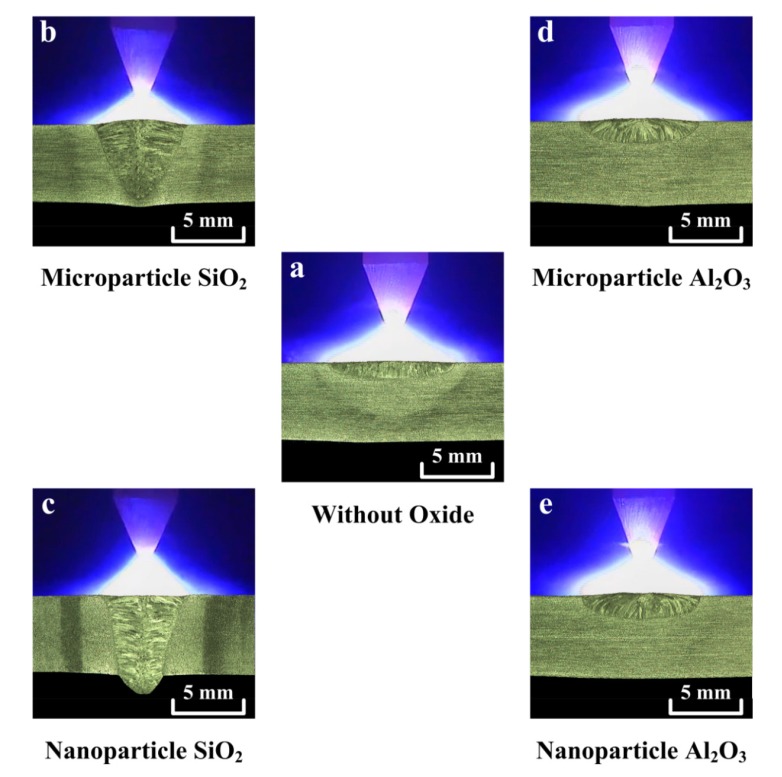
Weld shape and arc plasma of TIG welding with and without oxides.

**Figure 6:**
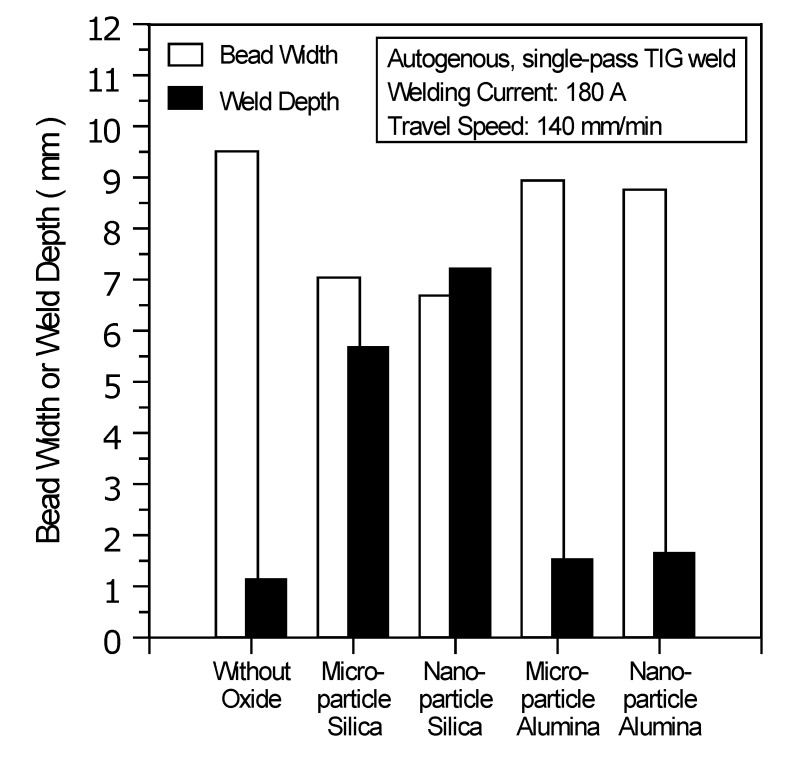
Geometric size of TIG welds made with and without oxides.

Various thermal stabilities and particle sizes of the activated flux have different effects on the fluid flow and heat transfer in the molten pool. These effects will greatly affect the shape and size of the weld. Figure 5 shows the weld shape and arc plasma of the TIG welding with and without oxides. The photograph is a combination of metallographic images (weld part) and CCD camera images (arc part). Figure 5a shows the result of C-TIG welding, which typically exhibits a wide, shallow weld. Figure 5b shows that use of the microparticle SiO_2_ results in a narrow, partial penetration weld. Figure 5c shows that the use of the nanoparticle SiO_2_ results in a narrow, complete penetration weld. As presented in Figure 5d,e, a wide, shallow weld was produced when using microparticle or nanoparticle Al_2_O_3_. The geometric shape of the TIG weld is strongly characterized by the weld depth and bead width. Figure 6 shows the geometric size of the TIG welds made with and without oxides. Compared with the C-TIG weld, there is an increase in weld depth and a decrease in bead width resulting from the use of microparticle or nanoparticle SiO_2_; however, the use of microparticle or nanoparticle Al_2_O_3_ results in no significant difference in weld depth and bead width.

Tseng *et al.* [4,5,6,7,8,10] reported experimental observations of an interactive mechanism between the arc column and the molten pool in A-TIG welding. Determining the joint penetration capability of the A-TIG weld can be conditionally subdivided into the mechanisms that take place in the arc column and the molten pool. In A-TIG welding, the arc column is characterized by the constricted arc plasma and the molten pool is characterized by the centripetal Marangoni convection. The mechanisms occurring in the arc column and the molten pool are interrelated and should be regarded as a unified system.

According to work by Lucas and Howse [22], the vaporized flux constricts the arc plasma by capturing electrons in the outer regions of the arc column. The attachment of electrons to vaporized molecules and dissociated atoms affect electron absorption, thereby forming negatively charged particles. As a result, restricting the current flow to the central region of the arc column increases the current density of the arc plasma and raises the arc temperature at the molten pool surface (anode spot). A-TIG welding provides a greater number of vaporized molecules and dissociated atoms in the outer regions of the arc column, and constriction of the arc plasma occurs as the number of electrons in the outer regions of the arc column significantly decreases. The reduction in the number of electrons is dependent on the electron affinity of an atom or molecule (defined as the change in energy of a neutral atom or molecule in the gaseous phase when an electron is added to the atom or molecule to form a negatively charged ion) in the outer regions of the arc column.

According to work by Heiple *et al.* [23,24,25], fluid flow in the molten pool determines the fusion zone geometry, and the dominant force that drives fluid flow is the temperature coefficient of surface tension (dγ/d*T*). If dγ/d*T* is non-zero in the molten pool, then fluid flows from regions of lower surface tension to regions of higher surface tension. Furthermore, dγ/d*T* is dependent on the oxygen content in the molten pool. In the TIG welding without activated oxide, dγ/d*T* in the molten pool generally has a negative value, resulting in centrifugal Marangoni convection. In the TIG welding with activated oxide, a certain amount of oxygen from the decomposition of activated oxide induces the centripetal Marangoni convection by inverting the dγ/d*T* in the molten pool. Lu *et al.* [26] showed that when the oxygen content in the stainless steel TIG weld metal was in the range of 70–300 ppm, the value of dγ/d*T* in the molten pool was positive; otherwise, the value of dγ/d*T* was negative.

**Figure 7:**
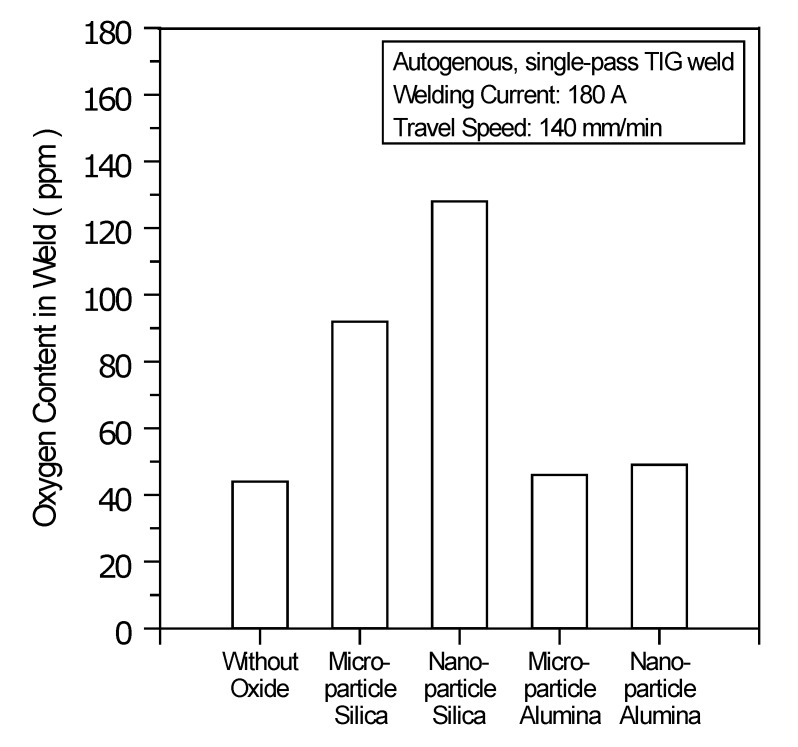
Oxygen content in TIG welds made with and without oxides.

In the TIG welding with SiO_2_, the number of electrons in the outer regions of the arc column may be reduced, because the Si molecule has a higher electron affinity (1.385 ± 0.005 eV [27]), and thus, the arc plasma is constricted (Figure 5b,c). The constricted arc plasma increases the current density of the arc plasma and generates a high arc heat at the anode hotspot. The resulting large Lorentz force contributes a forceful downward flow of arc heat within the molten pool. Moreover, according to the Ellingham diagram, SiO_2_ has a lower thermal stability than Al_2_O_3_. Figure 7 shows the oxygen content in the UNS S31603 stainless steel TIG welds made with and without oxides. The results show that the oxygen content in the stainless steel TIG weld metal made with microparticle SiO_2_ is 92 ppm, resulting in a sufficient oxygen content (> 70 ppm) for the molten metal, thus causing the Marangoni convection to reverse and move inward along the molten pool surface. The centripetal Marangoni convection further promotes the energy transfer of the arc heat from the surface to the bottom of the molten pool, resulting in a narrow, deep weld. Compared with microparticle SiO_2_, nanoparticle SiO_2_ has greater efficiency in improving the joint penetration capability of the A-TIG weld. In TIG arc heating and vaporization, the thermal dissociation and decomposition of nanoparticle SiO_2_ may occur much more readily than do the thermal dissociation and decomposition of microparticle SiO_2_. This results in the maximum capability of the joint penetration in the A-TIG weld. The TIG welding with nanoparticle SiO_2_ can result in a 524% increase in weld depth relative to the depth of the C-TIG weld.

Under the same welding conditions, the TIG welding with Al_2_O_3_ appears to have little effect on the arc column and the molten pool. In the TIG welding with Al_2_O_3_, the number of electrons in the outer regions of the arc column cannot be significantly reduced, because the Al molecule has a lower electron affinity (0.442 ± 0.010 eV [27]), and thus, the arc plasma cannot be constricted (Figure 5d,e). In addition, Al_2_O_3_ exhibits high thermal stability and possesses greater resistance to decomposition at high temperature. The oxygen content in the stainless steel TIG weld metal made with microparticle Al_2_O_3_ is 46 ppm, resulting in an insufficient oxygen content (< 70 ppm) in the molten metal and leaving the direction of Marangoni convection in the molten pool unchanged. Because Al_2_O_3_ could not make a positive contribution in constricting the arc plasma and inverting the Marangoni convection, The TIG welding with Al_2_O_3_ does not promote an increase in weld depth and a decrease in bead width. Moreover, the use of nanoparticle Al_2_O_3_ does not lead to a significant improvement in joint penetration capability compared with the capability achieved with microparticle Al_2_O_3_.

In arc welding, the power density of the welding heat source can be characterized by the D/W ratio, cross-sectional area and HAZ of the weld. Table 5 shows the D/W ratio, cross-sectional area and HAZ of the TIG welds made with and without oxides. Compared with C-TIG weld, the TIG weld made with microparticle or nanoparticle SiO_2_ leads to a high D/W ratio, large cross-sectional area and narrow HAZ; however, there is no significant improvement in the D/W ratio, cross-sectional area or HAZ resulting from the use of microparticle or nanoparticle Al_2_O_3_. A high D/W ratio, large cross-sectional area and narrow HAZ of the weld are characteristic of the increased power density of the heat source used in welding, which results in a high-energy density arc jet. Compared with microparticle SiO_2_, nanoparticle SiO_2_ produces a higher D/W ratio, larger cross-sectional area and a narrower HAZ of the A-TIG weld, resulting in a stronger, more forceful arc jet that has higher energy density. However, the use of nanoparticle Al_2_O_3_ does not lead to an enhancement of the energy density of TIG welding arc, unlike the use of nanoparticle SiO_2_.

**Table 5:** Depth-to-width (D/W) ratio, cross-sectional area and heat-affected zone (HAZ) of TIG welds made with and without oxides.

Characteristics	Activated Oxide				
	Without oxide	Microparticle SiO_2_	Nanoparticle SiO_2_	Microparticle Al_2_O_3_	Nanoparticle Al_2_O_3_
D/W ratio	0.12	0.81	1.08	0.17	0.19
Cross-sectional area	9.72 mm^2^	26.11 mm^2^	29.07 mm^2^	12.48 mm^2^	13.30 mm^2^
HAZ	1.72 mm	0.85 mm	0.60 mm	1.51 mm	1.42 mm

### 3.4. Effect of Microparticle or Nanoparticle Oxide on Angular Distortion of A-TIG Weldment

**Figure 8:**
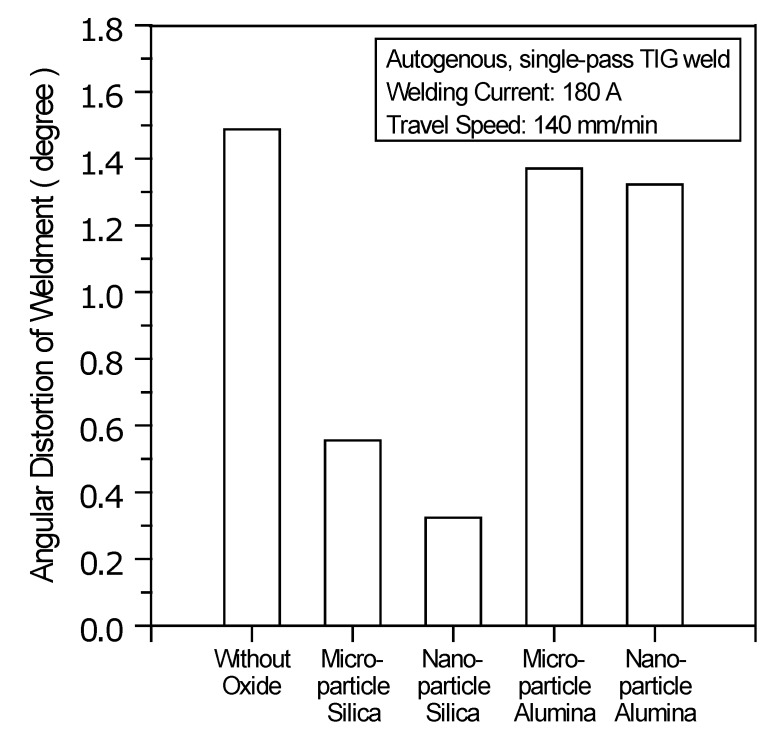
Angular distortion of TIG weldments made with and without oxides.

Angular distortion is one of the most common types of out-of-plane deformations. Angular distortion often occurs in a butt joint when transverse shrinkage is not uniform in the thickness direction of the welded plate [28,29,30]. Generally, the weld-induced distortion not only degrades the performance of the weldments, but also increases the rectification cost of the welded constructions. An investigation into the angular distortion of the A-TIG weldment is necessary in order to minimize weld-induced distortion. After the weldments were cooled to room temperature, all experiments were carried out to measure the angular distortion caused by welding. Figure 8 shows the angular distortion of the TIG weldments made with and without oxides. The results show a significant reduction in the angular distortion of the TIG weldment made with SiO_2_. In contrast, the TIG weldment made with Al_2_O_3_ shows no significant reduction in angular distortion compared with the C-TIG weldment.

In the case of TIG welding, the energy generated by the heat source is distributed in two ways; a portion is lost to the environment, and the remainder is transferred to the base metal [31]. The net energy of the arc heat delivered to the base metal is also distributed in two ways; a portion is used for fusion of the base metal, while the remainder is dissipated into the HAZ by heat conduction [32]. Therefore, a relatively small portion (maximum value of 0.35) of the net energy of the arc heat is used for the fusion of the base metal [31]. The arc heat dissipated into the HAZ causes a non-uniform thermal stress. Consequently, distortions are permanently produced in the weldment. A high power density of the welding heat source can significantly reduce arc heat loss to the HAZ, resulting in low thermal stresses acting on the weld metal and the adjacent HAZ.

### 3.5. Effect of Microparticle or Nanoparticle Oxide on Metallurgical Properties of A-TIG Weld Metal

Lippold and Savage indicated that the austenitic stainless steel weld metals, which exhibit delta ferrite content of 5–12 FN provided the best resistance to hot cracking [33]. It is necessary to attain the desired levels of delta ferrite content in the UNS S31603 stainless steel A-TIG weld metal.

**Figure 9:**
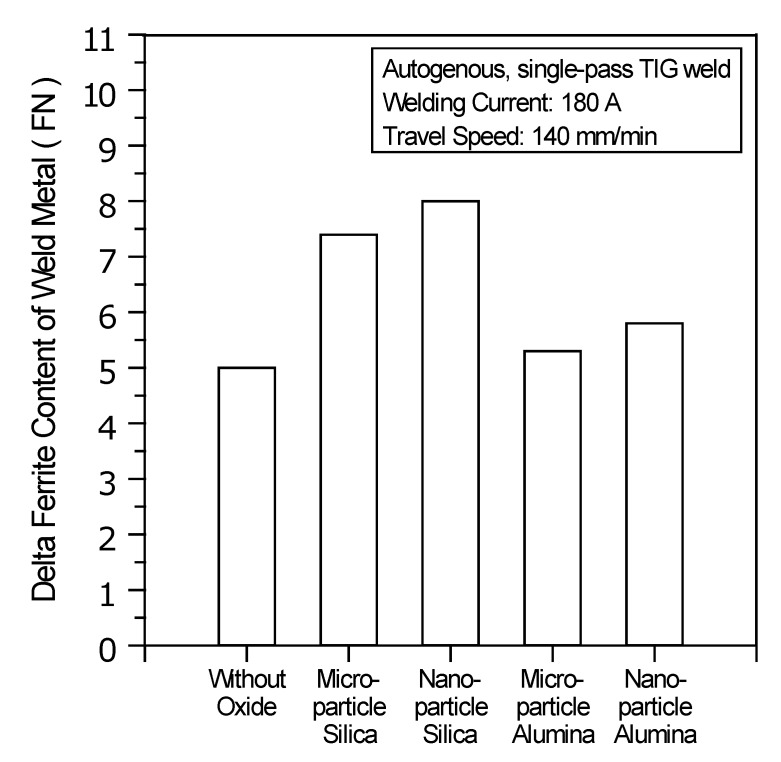
Delta ferrite content of TIG weld metals made with and without oxides.

Figure 9 shows the delta ferrite content of the UNS S31603 stainless steel TIG weld metals made with and without oxides. The base metal used in this study was hot-rolled stainless steel having an ultra-low ferrite content of 0.4 FN. In the stainless steel C-TIG weld metal, the ferrite content was increased to 5.0 FN from its initial value of 0.4 FN. This is because the UNS S31603 stainless steel weld metal solidified in the ferritic-austenitic (FA) mode, which has delta ferrite as the primary phase. During welding, the cooling rate of the weld metal is so rapid, that phase transformation of the delta ferrite to the austenite is not complete, resulting in more delta ferrite being retained in the stainless steel weld metal after solidification.

The delta ferrite content of the UNS S31603 stainless steel TIG weld made with SiO_2_ is in the range of 7.4–8.0 FN; with the use of Al_2_O_3_, the ferrite content is in the range of 5.3–5.8 FN. The power density of the welding heat source directly affects the amount of arc heat supplied for the fusion of the base metal and affects the formation of the delta ferrite phase in the weld metal. An increase in the power density decreases the arc heat required for the fusion of the base metal, because it decreases the time required for using the heat source for fusing the base metal. Compared with C-TIG welding, the TIG welding with SiO_2_ uses a heat source with a higher power density, resulting in a higher ferrite content in the weld metal, as the transformation of delta ferrite to the austenite phase has less time to occur at a high cooling rate. However, the use of microparticle or nanoparticle Al_2_O_3_ does not lead to a significant increase in ferrite content, in contrast to welding without any oxide.

**Figure 10:**
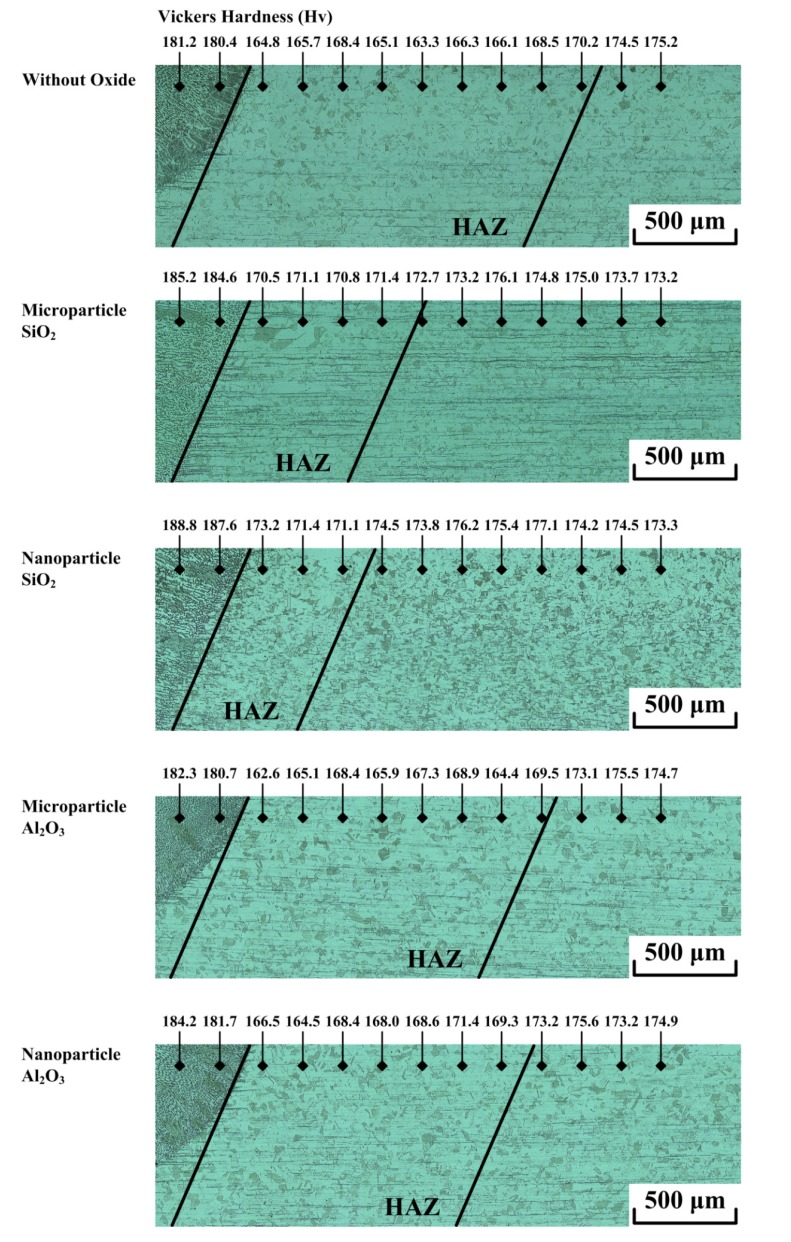
Hardness distribution of TIG weldments made with and without oxides.

Figure 10 shows the hardness distribution of the TIG weldments made with and without oxides. The results show that the weld metal has the highest Vickers hardness, whereas the HAZ has the lowest Vickers hardness. In the UNS S31603 stainless steel TIG welding with or without oxide, higher hardness is achieved by forming microstructures that consist of the delta ferrite in the weld metal; lower hardness is associated with a relatively coarse austenite grain size in the HAZ. As previously mentioned, the TIG weld metal made with SiO_2_ has a higher delta ferrite content, resulting in a higher Vickers hardness compared to that of the C-TIG weld metal or the TIG weld metal made with Al_2_O_3_. The results also show that the use of nanoparticle oxide does not lead to a significant increase in Vickers hardness compared with that achieved with microparticle oxide. Furthermore, the lower power density of the heat source for C-TIG welding or the TIG welding with Al_2_O_3_ requires a larger amount of arc heat for fusion of the base metal, resulting in a greater amount of arc heat being dissipated to the HAZ. The HAZ thus has a coarser austenite grain than that of the base metal, which tends to decrease the hardness of the HAZ. Compared with C-TIG welding or the TIG welding with Al_2_O_3_, the TIG welding assisted by SiO_2_ uses a heat source with a higher power density. Therefore, a smaller amount of the arc heat dissipates to the HAZ, resulting in only a slight decrease in the hardness of the HAZ compared to that of the base metal. In conclusion, the use of nanoparticle oxide results in no significant difference in the delta ferrite content and Vickers hardness compared with the weld metal made with microparticle oxide or without any oxide.

## 4. Conclusions

This study has demonstrated that the performance of the UNS S31603 stainless steel A-TIG weld greatly depends on the thermal stability and the particle size of the activated oxides. The relationship between the activated oxide and polar solvent in the A-TIG welding was also investigated. The main conclusions obtained from this study are as follows:

(1) Water or methanol produces a homogeneous distribution of the flux-coated layer. Compared with microparticle oxide, nanoparticle oxide mixed with water or methanol provides good coverability of the coated layer. However, this is accompanied by poor flux spreadability.
(2) Compared with Al_2_O_3_, SiO_2_ produces a satisfactory surface appearance of the A-TIG weld. The surface appearance of the TIG weld made with nanoparticle oxide has less flux slag compared with the one made with microparticle oxide of the same type.
(3) In TIG arc heating and vaporization, thermal dissociation and decomposition of nanoparticle SiO_2_ may occur much more readily than do the thermal dissociation and decomposition of microparticle SiO_2_. This results in the maximum joint penetration capability of the A-TIG weld. The TIG welding with nanoparticle SiO_2_ can result in a 524% increase in weld depth relative to the depth of the C-TIG weld. However, the use of nanoparticle Al_2_O_3_ does not lead to a significant increase in the joint penetration capability of the A-TIG weld.
(4) Compared with microparticle SiO_2_, nanoparticle SiO_2_ produces a higher D/W ratio, larger cross-sectional area and narrower HAZ of the A-TIG weld. This results in minimum angular distortion of the A-TIG weldment. Compared with the angular distortion of the C-TIG weldment, the TIG welding with nanoparticle SiO_2_ can result in a 78% reduction in angular distortion. In contrast, the TIG weldment made with nanoparticle Al_2_O_3_ shows no significant reduction in angular distortion.
(5) Compared with C-TIG welding, the TIG welding with microparticle or nanoparticle SiO_2_ has a higher delta ferrite content, resulting in a higher Vickers hardness in the UNS S31603 stainless steel TIG weld metal. In contrast, microparticle or nanoparticle Al_2_O_3_ results in no significant difference in metallurgical properties (ferrite content and hardness) compared to that of the C-TIG weld metal.
(6) In the case of the UNS S31603 stainless steel TIG welding assisted by activated oxide, the thermal stability of the oxide plays a significant role in enhancing the capability of the joint penetration compared with the oxide particle size.
